# Validation of the Portuguese version of the diabetes self-management questionnaire-revised (DSMQ-R) in people with type 2 diabetes mellitus

**DOI:** 10.1186/s40359-024-01900-9

**Published:** 2024-07-23

**Authors:** Dulce Oliveira, Andreia Costa, Adriana Henriques, Maria Alice Curado, Andreas Schmitt, Paulo Nogueira

**Affiliations:** 1 Nursing Research, Innovation and Development Centre of Lisbon (CIDNUR), School of Nursing of Lisbon (ESEL), Lisbon, Portugal; 2 Local Health Unit of Amadora/Sintra, E.P.E., Primary Health Care of Amadora, Amadora, Portugal; 3 Istituto de Saúde Ambiental (ISAMB), Lisbon University, Lisbon, Portugal; 4 School of Nursing of Lisbon (ESEL), Lisbon, Portugal; 5 Diabetes Center Mergentheim, Research Institute Diabetes Academy Mergentheim, Bad Mergentheim, Germany

**Keywords:** Type 2 diabetes mellitus, Measurement properties, Self-care behaviour, HbA1c, Cultural validation

## Abstract

**Background:**

Reflecting people with diabetes’ self-management activities is often required in both research and clinical practice. This study evaluated the measurement properties of the Portuguese version of the Diabetes Self-Management Questionnaire-Revised (DSMQ-R) on a sample of people with type 2 diabetes mellitus (T2DM).

**Methods:**

Translation and cultural adaptation were conducted according to guidelines for cross-cultural adaptation and validation of healthcare measurement instruments. A cross-sectional study was performed including 365 people with T2DM in primary care. Reliability, construct validity, and criterion validity were analyzed.

**Results:**

The total scale of the translated DSMQ-R revealed sufficient internal consistency (alpha = 0.82), and most of the subscales performed adequately. The exploratory factor structure was robust, and confirmatory analysis showed a good model fit with the scale structure of the original scale. The scale scores correlated with the participants’ last HbA1c estimates, supporting convergent validity, and convergence was confirmed by the adequate average variance extracted.

**Conclusions:**

The Portuguese version of the DSMQ-R is a reliable and valid tool for gauging self-management behaviors in people with T2DM and their relationship with glycemic values.

## Background

The global prevalence of diabetes mellitus has increased, impacting individuals at progressively younger ages and incurring substantial societal costs [1]. Among European countries, Portugal has an average diabetes incidence of 14.1%. Recent findings from the National Diabetes Observatory indicate that by 2021, more than one million Portuguese individuals aged between 20 and 79 years were diagnosed with some type of diabetes [2]. Factors contributing to this negative upward trend include adverse lifestyle factors such as physical inactivity, unhealthy food and dietary behaviors, smoking, overweight, and obesity, which increase the risk of developing T2DM [3–5].

Diabetes is a chronic disease swayed by a mixture of psychosocial, behavioral, and metabolic factors that are considered fundamental for its management and progression [6]. It´s selfmanagement is a complex, dense, dynamic, and continuing process that demands a person’ active role, with a constant drift of new knowledge, medication strategies, and behaviour changes [7–10]. The burden of disease management demands substantial lifestyle transformations and coping processes that can have an impact on a person’s daily routines and quality of life, compromising personal care and potentially leading to states of anxiety, stress, or even depression [11]. Effective diabetes self-care behaviour is mandatory and essential for achieving optimal glycemic control and preventing acute and long-term complications of T2DM [12]. Persistent hyperglycemia can lead to serious long-term complications such as retinopathy and vision impairment, neuropathy, coronary heart disease, and chronic kidney disease impacting the quality of life of a person [13]. Continually elevated HbA1c levels are associated with significantly increased morbidity and mortality risks [14]. Adopting diabetes self-care behaviors that promote good metabolic management is crucial for preventing metabolic imbalances’ progression and detrimental effects [15]. Individuals are tasked with adhering to recommendations for optimal self-care behaviour and particular medical treatment regimens ranging from maintaining a balanced, fat- and glucose-oriented diet and regular physical activity to routine glucose checks, consistent medication taking, and recognizing and treating hyper and hypoglycaemic symptoms [16, 17]. Research has shown that better self-care behaviors are related to better HbA1c levels and better metabolic control [3, 5, 18, 19]. Given the indispensable role of daily self-care in diabetes management, the need for dependable and precise instruments for its assessment is evident [20].

The Diabetes Self-Management Questionnaire-Revised DSMQ-R is an instrument that provides a multidimensional assessment of self-care activities relevant to achieving good glycemic levels [21] and is recommended for the assessment and production of true results [21, 22]. According to the Consensus-based Standards for the Selection of Health Measurement Instruments (COSMIN), which allows us to choose the most appropriate instrument for measuring an outcome, the DSMQ-R may be recommended as an instrument for assessing self-care behaviors in people with T2DM [21, 22].

In light of these considerations, this study aimed to adapt and appraise the psychometric attributes of the DSMQ-R for Portuguese people with type 2 diabetes mellitus.

## Subjects, methods and materials

### Description of the DSMQ-R

The DSMQ-R assesses diabetes self-care behaviors relevant to glycemic management using 20 core questions plus 7 optional questions. The original German instrument has been translated and culturally adapted into several languages, including English, Indian, Malay, Urdu, Chinese, Spanish, Romanian, Arabic, and Turkish. The twenty mandatory questions assess self-care behaviors that can be important or useful for people with both T2DM and T1DM; the seven additional questions focus on behaviors relevant to intensive insulin treatment regimens (i.e., basal and bolus insulin with multiple daily injections or insulin pump therapy); thus, their application is optional. Each question is scored on a four-point Likert scale from 0 to 3 (0 - Does not apply to me, 1 - Applies to me to some degree, 2 - Applies to a moderate degree, and 3 - Applies to me very much).

All 27 questions were translated and culturally adapted, but only the first twenty questions were analysed for psychometric properties, as the subsample of patients with T2DM receiving intensive insulin treatment collected in this study was too small. The final score of the questionnaire, which is made up of positive and negative questions, can be calculated using the scores of all twenty questions or using the subscales when the assessment is intended to evaluate specific behaviors or dimensions of self-care. The four main subscales are Eating behavior (items 2, 5, 9, 13, 17, and 18), Glucose management (items 1, 4, 6, 10, and 12), Physical activity (items 8, 11, and 15), and Cooperation with the healthcare team (items 3, 7, 14, and 19). The Glucose management scale comprises questions on Medication-taking and glucose measurement behaviors and thus may be subdivided into a Medication-taking subscale (items 4 and 12) and a Glucose monitoring subscale (items 1, 6, and 10). The item, values are summed and transformed to scale scores ranging between 0 and 10, with higher values indicating better behavior.

### Translation and adaptation of the DSMQ-R

The translation and cultural adaptation of the DSMQ-R instrument from English were carried out by respecting semantic equivalence and adapting the lexicon to the culture of the Portuguese population and following the guidelines for translation and back-translation of a healthcare instrument methodically and rigorously [23, 24]. The forward translation process began with a first translation into Portuguese, which was carried out by two independent bilingual Portuguese translators with knowledge of diabetes terminology and linguistic and cultural details. An independent third reviewer compared version 1 and version 2 and discussed divergences with the research team, leading to a preliminary initial version. A blind independent backwards translation was then carried out by 2 independent translators, one of whom was a native English speaker. Both translators had no prior knowledge of the original instrument or its objectives. The two versions obtained were compared and analysed by a group of experts, resulting in the final version. The final version was pilot-tested on twenty people with T2DM (10 with and 10 without intensive insulin treatment). After the pilot test, the group of experts analysed the conceptual and contextual equivalences, obtaining the final Portuguese version of the instrument.

### Study design

A cross-sectional study was carried out between September 2022 and March 2023. The basis for calculating the sample was a population of more than 1.600 people with T2DM attending diabetes nursing appointments at a primary healthcare unit in the Lisbon metropolitan area, Portugal. The sample size was calculated using Survey Monkey^®^ software [25], with an estimated 95% confidence interval (CI), a standard deviation (SD) of 1.96, and a 5% margin of error. The results of the sample calculation suggested recruiting 320 people, but assuming that there could be barriers to recruiting and retaining the sample, the number of people to recruit was increased by 20%, with a final total of 365 participants recruited.

Recruitment was carried out by five nurses via either face-to-face or telephone contact, yielding a convenience sample. All patients over the age of 18 and with a diagnosis of T2DM were invited to participate in the study; those with insufficient Portuguese language skills, which would compromise the interpretation of the questionnaire, were excluded. Eligible people were informed about the study aims and procedures. All study participants provided written informed consent.

### Sociodemographic, anthropometric, and clinical data

Sociodemographic and clinical data were self-reported by the participants or documented by the nurse using the participants’ clinical files (e.g., BMI, HbA1c, known duration of diabetes, number, and type of diabetes complications).

### Statistical analyses

Descriptive, inferential, and multivariate statistical analyses were carried out using IBM SPSS Statistics 29.0 for Windows [26], and confirmatory factor analysis was performed using the JAMOVi software package 2.3.26 [27]. The Kolmogorov‒Smirnov test was used to assess the assumption of normality of distribution. The statistical significance level was set at a p-value ≤ 0.05.

Reliability was assessed to measure the instrument’s ability to reproduce consistent results over time by two reliability criteria, stability and internal consistency. The instrument was initially administered for the first time to 365 participants in the study and then repeated after two weeks to 50 participants in the sample [24]. The test-retest method was used to assess the instrument’s stability over time by calculating the intraclass correlation coefficient (ICC). The instrument’s internal consistency was assessed using Cronbach’s α of the sum scale, which included all the items and the specific subscales.

The instrument’s validity was assessed for construct validity using exploratory factor analysis (EFA) and confirmatory factor analysis (CFA), discriminant validity via known-groups analysis, and convergent validity through convergent correlations with HbA1c as criterion analysis.

The analysis used the results of the 365 people with T2DM to carry out EFA. As a precondition for the EFA, Barlett’s test of sphericity was carried out, and the Kaiser‒Meyer‒Olkin (KMO) sampling adequacy value was obtained. KMO values ˃ 0.5 and Bartlett’s test of sphericity with pvalue˂0.05 were considered good for proceeding with the EFA [28–30]. EFA was carried out using the principal axis factoring method with oblimin rotation. Factors to be retained were determined according to the Kaiser criterion (requiring factor eigenvalues > 1) [30].

To perform CFA, a subsample was selected randomly based on a ratio of 10 participants for each item, [31]. Using CFA, the quality of fit of the model proposed by the EFA matrix and the validity of the model were evaluated. As an indicator of model fit, the following reference values were used for the fit indices: normed chi-square (X2/g. l) ˂ 2, suggesting good fit; comparative fit index (CFI) and Tucker‒Lewis index (TLI) ≥ 0.95, suggesting good fit; goodness fit index (GFI) ≥ 0.95, suggesting very good fit; and root mean square error of approximation (RMSEA) ˂ 0.05, suggesting very good fit. The values of the items suggested in the EFA with factor loadings ˂ 0.3 were not included in the CFA.

Discriminant validity was assessed via known-groups analysis testing the instrument’s ability to distinguish and discriminate between different groups of the variable of interest [32]. Thus, three groups were established according to HbA1c values, with ≤ 7.5% (58.5 mmol/mol) considered “good”, between 7.6% and 8.9% (60–74 mmol/mol) considered “suboptimal” and 9.0% (75 mmol/mol) considered “in need of improvement”. HbA1c values were associated with metabolic control and were related to the group’s performance on the DSMQ-R sum scale and subscales. The differences between the groups were tested for significance using ANOVA.

The assessment of the instrument’s convergent validity makes it possible to establish the degree of correlation between a measure and the instrument’s construct. The criteria used to determine convergence were factor loading, composite reliability, and average variance extracted (AVE). For a convergent model, values of AVE ˃ 0.5 and values of AVE ˂ 0.5, but with a composite reliability > 0.6, according to Fornell-Larcker, were considered good [33].

## Results

### Sample characteristics

A total of 365 people with T2DM participated in the study. The sample characteristics are shown in Table 1. The distribution of sex was balanced (51.2% were men), and the average age was 67 (± 11) years. The mean BMI was 28.9 kg/m² (± 5.3). The participants reported a mean time since the T2DM diagnosis of 11 (± 6.5) years. The mean HbA1c was 7.3 (± 1.8) (56.3 mmol/mol/±4.0). Oral antidiabetic drugs were the most frequent medical therapy used by 76.4% of the sample; 13.7% (*n* = 50) used insulin, but only 22 used an intensive insulin regimen. 13% were diagnosed with diabetes complications.

**Table 1:** Characteristics of the study sample

Person variables	*n* = 365
Female sex	178 (48.8%)
Age	67 (± 11)
BMI (kg/m2)	28.9 (± 5.3)
Education	
Primary education (4 years)	192 (52.6%)
Lower secondary education (6 years)	63 (17.3%)
Upper secondary education (9 years)	29 (7.9%)
Postsecondary nontertiary education (12 years)	53 (14.5%)
Bachelor´s degree	23 (6.3%)
Master’s degree	5 (1.4%)
Occupation status	
Employed	81 (22.2%)
Self-employed	20 (5.5%)
Retired	238 (65.2%)
Unemployed	10 (2.7%)
Other	16 (4.4%)
Marital status	
Married	239 (65.5%)
Divorced	30 (8.2%)
Single	40 (11.0%)
Widowed	56 (15.3%)
HbA1c	
In %	7.3 (± 1.8)
In mmol/mol	56.3 (± 4)
Diabetes treatment	
Oral antidiabetic medication only	280 (76.7%)
Oral antidiabetic medication + insulin	35 (9.6%)
Insulin only	10 (2.7%)
Diet + exercise only (no medication)	27 (7.4%)
Injectable therapy (incretin mimetic/GLP1)	13 (3.6%)
Injectable therapy + insulin	5 (1.4%)
Years since diabetes diagnosis	10.89 (± 6.49)
Diabetes complications	
With any diabetes complications	48 (13.2%)
Diabetic retinopathy	23 (6.3%)
Diabetic nephropathy	18 (4.9%)
Diabetic neuropathy	2 (0.6%)
Foot ulcer	16 (4.4%)

*Note* Data are n (%) or M (± SD)

### DSMQ-R total and subscale scores

The Portuguese sample with T2DM had a mean total score of 6.5 (± 1.4) on the DSMQ-R sum scale out of a possible maximum of 10 points (Table 2). The subscale scores were as follows: Eating behavior = 8.1 (± 1.7), Medication taking = 8.6 (± 2.3), Glucose monitoring = 6.6 (± 3.1), Physical activity = 5.3 (± 2.7), and Cooperation with the healthcare team = 6.5 (± 1.7) (Table 2).

**Table 2:** Scale and subscale characteristics of the Portuguese version of the DSMQ-R and reliability indices (*n = 365*).

	M (±SD)	Internal consistency (α)
**DSMQ-R Scale**	6.54 (± 1.4)	0.82
DSMQ-R Scale per item	-	-
1. I check my blood sugar levels (glucose levels) with care and attention.	2.25 (± 0.8)	-
2. The foods I choose to eat make it easy for me to achieve good blood sugar levels.	1.93 (± 0.6)	-
3. I regularly see the doctor (diabetes specialist) regarding my diabetes.	2.70 (± 0.6)	-
4. I take my diabetes medication (e.g. insulin, tablets) as prescribed/agreed.	2.72 (± 0.7)	-
5. Occasionally I eat lots of sweets or other foods rich in carbohydrates.	1.71 (± 0.7)	-
6. I keep records of my blood sugar values (or CGM data) to better manage my diabetes.	2.08 (± 0.9)	-
7. I tend to avoid seeing the doctor (diabetes specialist) regarding my diabetes.	2.60 (± 0.8)	-
8. I am regularly physically active to improve my diabetes/my health.	1.51 (± 0.9)	-
9. I follow the relevant dietary recommendations for people with diabetes (e.g. given to me by my doctor or diabetes specialist).	2.01 (± 0.7	-
10. I do not check my blood sugar levels (glucose levels) frequently enough to achieve good glucose control.	2.10 (± 1.0)	-
11. I avoid physical activity although it would be good for my diabetes/my health.	1.86 (± 1.0)	-
12. I tend to forget or skip my diabetes medication (e.g. insulin, tablets).	2.55 (± 0.9)	-
13. Sometimes I have real ‘food binges’ (not triggered by hypoglycaemia).	2.18 (± 0.9)	-
14. Regarding my diabetes, I should see my doctor (diabetes specialist) more often	1.93 (± 1.0)	-
15. I am less physically active than would be optimal for my diabetes/my health.	1.40 (± 1.0)	-
16. I could improve my diabetes self-care considerably.	1.42 (± 0.8)	-
17. I estimate the carbohydrate content of my meals (to achieve better glucose control).	0.62 (± 0.8)	-
18. I eat without regard to my diabetes.	1.87 (± 0.9)	-
19. I check/discuss my diabetes treatment with the doctor (diabetes specialist) regularly.	2.17 (± 0.9)	-
20. My diabetes self-care is poor.	1.89 (± 0.9)	-
**Eating Behavior Subscale**	8.07 (± 1.7)	0.62
**Glusose Managment Subscale**	7.82 (± 2.0)	0.82
Glusose Monitoring subscale	6.63 (± 3.1)	0.97
Medication-Tacking subscale	8.59 (± 2.3)	0.68
**Physical Activity subscale**	5.26 (± 2.7)	0.72
**Cooperation with the Healthcare Team subscale**	6.54 (± 1.7)	0.66

*Note* Data are M (± SD) and Cronbach’s (α)

### Reliability analyses

The test-retest intraclass correlation coefficient (ICC) was 0.96 (p˂0.001), indicating that the instrument’s measurement had excellent stability over two weeks [34]. Analysing the internal consistency of the 20-item scale by Cronbach’s α revealed a coefficient α of 0.82, which is considered to reflect moderate to high reliability. The coefficients for the subscales were 0.97 for Glucose monitoring, 0.68 for Medication taking, 0.62 for Eating behavior, 0.72 for Physical activity, and 0.66 for Cooperation with the healthcare team (Table 2). The correlation between items was assessed concerning the total scale and subscales. According to the evaluation of changes in α, if items were deleted, deletion of item, 17 would increase Cronbach´s α DSMQR total scale to 0.83, but not significantly, and the Eating Behaviour subscale to 0.70. During the reliability analysis of the subscales, the different items were analysed. The study of the other subscales showed no improvement with the possible deletion of items; for certain subscales that are already part of a subscale, such as the Medication Counselling subscale, the deletion of items would not be possible, since they only consist of two items.

### Validity analyses

#### Construct validity

The Kaiser–Meyer–Olkin (KMO) value was 0.81, indicating good sampling adequacy, and Bartlett’s test of sphericity indicated that the correlations between the items were good (*p* < 0.001).

The initial exploratory factor analysis of the 20 items, using the principal axis factoring method with oblimin rotation, indicated that six factors fulfilled the Kaiser criterion (eigvalue˃1), explaining 50.1% of the variance in the data set. It had the following structure: Factor 1 with items 1, 6, and 10; Factor 2 with items 2, 5, 7, 9, 13, 14, 16, 18, and 20; Factor 3 with items 8, 11, and 15; Factor 4 with items 3 and 19; Factor 5 with items 4 and 12; and Factor 6 with items 14 and 17. Only statistically significant factor loadings with values ≥ 0.30 were considered, so item, 7 was removed from the analysis. A subsequent exploratory analysis excluded item, 14, which was saturated in more than one factor (factor 2 and factor 6), and item, 17, which was retained alone in factor 6. Following the analysis, item, 16 was removed due to its high modification index values. The final EFA model of 5 factors maintained a good explanation of the variance in the data set, with a slight increase to 52.7%, and each factor extracted explained at least 5% of the total variance [30]. Factor 1, composed of items 1, 6, and 10 (Glucose monitoring), explained 15.8% of the total variance; factor 2, with items 2, 5, 9, 13, 18, and 20 (Eating behavior), explained 12.7%; factor 3, composed of items 8,11, and 15 (Physical activity), explained 9.8%; factor 4, including items 4 and 12 (Medication taking), explained 8.0%; and factor 5, with items 3 and 19 (Cooperation with the healthcare team), explained 6.4%.

**Fig. 1:**
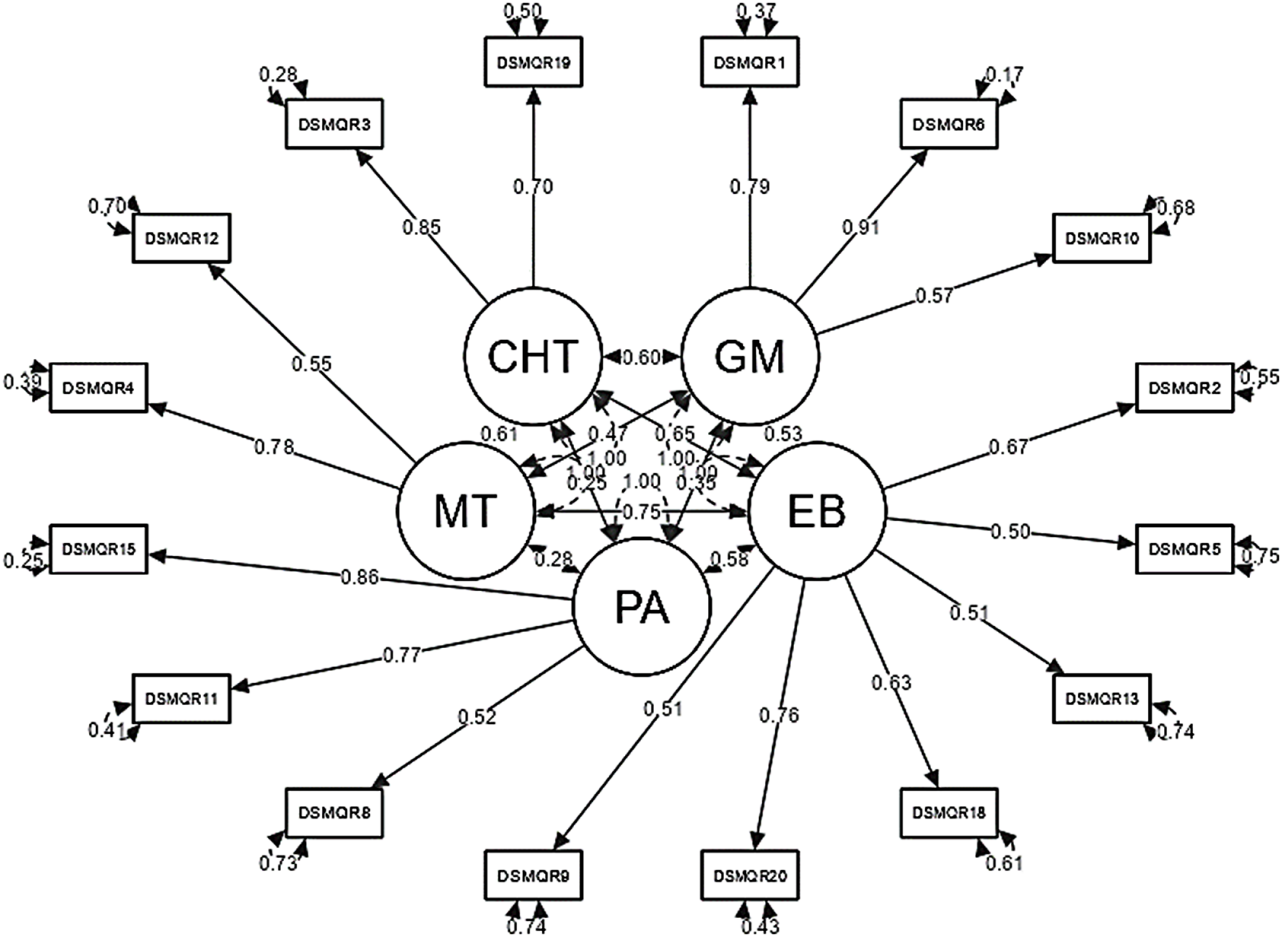
Confirmatory five-factor model of the DMSQ-R displaying the individual reliability of each item, and residual. *Notes*: Data are factor loadings (one-headed arrows), correlations between factors (two-headed arrows) or residuals. Ovals indicate latent variables (factors), boxes indicate manifest measurement variables (questionnaire items). GM = glucose monitoring; MT = medication tacking; EB = glucose behavior; CHT = cooperation with the healthcare team; PA = physical activity

CFA was then carried out using a random subsample of 10 participants for each variable to test the expected factor structure in line with the scale structure being analysed (model displayed in Fig. 1). The model, using the analysed sample of 200 participants, showed an excellent fit to the data (*X*^*2*^ (1.65) ˂0.001; CFI = 0.989; TLI = 0.986; GFI = 0.979; RMSEA = 0.039 (95% CI 0.014–0.057]). All the loadings presented were significant at *p* < 0.05. The complete CFA results are given in Fig. 1.

#### Convergent validity

The convergent validity of the different subscales was adequate, with an average variance extracted (AVE) of ˃ 0.50. The Eating Behavior subscale showed a lower average variance extracted (0.40) but had a composite reliability of 0.67, which supports the adequate convergence of the subscale (Table 3).

**Table 3:** Composite Reliability and average variance extracted from the DSMQ-R total scale and subscale (*n = 365*)

	Composite Reliability (α)	Factor Loading (ω)	Average Variance Extracted
Eating Behavior	0.67	0.68	0.40
Glusose Monitoring subscale	0.87	0.86	0.76
Medication-Tacking subscale	0.54	0.60	0.60
Physical Activity	0.67	0.67	0.56
Cooperation with the Healthcare Team	0.58	0.64	0.60

*Note* Data are Cronbach’s α, McDonald’s omega and average variance extracted (AVE)

#### Known-group validity

The known-groups assessment showed that the group with in-target metabolic control (HbA1c ≤ 7.5%, 58.5 mmol/mol) had better self-care values than those outside the target range (HbA1c ≥ 9.0%, 75 mmol/mol). When the subscales were compared, the trend was maintained. The same can be observed between the groups with HbA1c values ≤ 7.5% (58.5 mmol/mol) compared to 7.6 – 8.9% (60–74 mmol/mol), except for the Glucose Monitoring subscale, where the difference was not significant (*p* = 0.823) (Table 4).

**Table 4:** Assessment of the DSMQ-R self-care status according to HbA1c ≤ 7.5%, 7.6–8.9%, and ≥ 9.0% among known groups (*n = 365*)

	HbA1C ˂7.5% (58.5 mmol/mol)	HbA1C 7.6 – 8.9% (60–74 mmol/mol)	HbA1C ˃ 9.0% (75 mmol/mol)	*P*-value
DSMQ-R Sum Scale	6.7 ±1.4	6.3 ±1.3	5.8 ±1.7	0.000
Eating Behavior	8.2 ± 1.6	7.7 ± 1.8	7.4 ± 1.7	0.003
Glusose Managment subscale	7.9 ±2.0	7.9 ± 1.7	7,1 ± 2.1	0.025
Glusose Monitoring subscale	6.7 ± 3.1	6.7 ±2.9	6.3 ±2.8	0.823
Medication-Tacking subscale	8.7 ± 2.3	8.5 ± 2.0	7.8 ± 2.6	0.037
Physical Activity	5.4 ± 2.7	5.0 ± 2.3	4.4 ± 2.6	0.042
Cooperation with the Healthcare Team	6.7 ± 1.4	6.1 ± 1.4	6.1 ± 1.5	0.011

*Note* Data are n (%) or M (± SD)

## Discussion

Maintained supported self-management in partnership with health professionals, with the (re)knowledge of a person´s needs, can help foster the necessary person´s self-confidence to take an active role in self-managing their illness and their health selfcare behaviours [35, 36]. To better understand how healthcare professionals can support people with T2DM in self-managing and preventing future complications, it is considered essential the apply assessment tools to find out what their care is in different areas [20].

The present study performed a psychometric analysis of the Portuguese version of the DSMQ-R, establishing the psychometric attributes of the instrument after undergoing translation, adaptation, and validation processes tailored to the Portuguese T2DM population [24]. The language of the translated instrument was clear and understandable to the targeted audience.

The sample under study showed an average level of self-care similar to studies carried out in Europe, as seen in the multicenter, cross-sectional German population [37], but lower compared to the cross-sectional cohort study carried out in the Hungarian population [38]. With an average score of 6.5 on a maximum scale of 10 points, we can see that there is still some way to improve the self-care management of the T2DM Portuguese sample.

In examining the reliability of the total scale, the study showed good retest reliability and internal consistency. Interestingly, the original DSMQ-R iteration for people with T2DM showed an alpha range between 0.84 and 0.89 [21], while some of its subscales presented lower Cronbach’s α values (in line with the number of items per scale). Three subscales, namely, Eating Behavior, Cooperation with the healthcare team, and Medication taking, reflected these lower values. The lower value in the Eating Behavior subscale could be attributed to item, 17 (*I estimate the carbohydrate content of my meals).* Item 17 was an item, added to the first version of the DSMQ (16 items) and is intended to assess glycemic control, especially in people who make insulin adjustments to manage glycemic control. In this study, as most of the population was not under intensive insulin treatment and thus was not receiving insulin adjustments, this item, was not answered by many participants. With the elimination of item 17, a slight improvement in the internal consistency of the DSMQR scale to 0.83 and a more consistent improvement in the Eating Behaviour subscale to 0.70 can be observed. Therefore, the elimination of item 17, could be considered in future interactions of the scale. Cooperation with the healthcare team was also identified in prior studies with lower Cronbach’s α values [38]. This could be related to questions 14 “*Regarding my diabetes*,* I should see my doctor (/diabetes specialist) more often*” (1.9 (± 1.0) and 19 “*I check/discuss my diabetes treatment with the doctor (/diabetes specialist) regularly*” (1.9 (± 0.9), which had lower values. These figures may reflect the post-COVID-19 pandemic period during which the questionnaires were administered. During the pandemic period, accessibility to healthcare and healthcare professionals in Portugal was suspended or reduced but then gradually resumed (Ferreira et al., 2023). The third item, with the lowest Cronbach’s α value, was the Medication Taking subscale made up of items 4 and 12, with a value of 0.68. This may be because this subscale only has two items with semantically equal questions, one positive and the other negative, as corroborated by Schmitt et al. for the T2DM population [21]. In the translation and validation study of the same scale for the Hungarian language [38], a decrease in internal consistency was attributed to item, 12 (*I tend to forget or skip my diabetes medication*), for having lower levels of self-care in adherence to the medication regimen, translated as forgetting or not taking the prescribed medication, which was also observed in this study. In other studies, the same behavior has also been observed for this subscale [37, 39]. The retest showed that the DSMQ-R is an instrument that remains stable over time and has high reproducibility, in line with previous studies [21].

The convergent validity of the instrument was confirmed through adequate average variance extracted ˃0.5 demonstrated in the DSMQ-R scale and most of the subscales: Glucose monitoring, Medication-tacking, Physical activity, and Cooperation with the healthcare team. Assessing known-group validity made it possible to assess the differences between groups based on HbA1c: people with HbA1c levels in the target range reported better DSMQ-R scores than did people in the other groups. This finding accentuated the correlation between enhanced self-care results and improved metabolic control, a pattern consistent with prior literature [15, 38, 40]. Among the subscales, physical activity scored the lowest and emerged as a self-care dimension with room for improvement. This finding aligns with Eurobarometer findings highlighting Portugal’s alarming high inactivity rates, with 73% of its population abstaining from regular exercise [41].

Furthermore, our exploratory factor analysis revealed that the extraction of five factors in line with the expected scale structure explained more than 50% of the variance in the data set, as required [30]. Some discrepancies emerged in the Portuguese DSMQ-R version of the scale concerning item, saturation, and modification indices suggested by some factors related to the original scale, such as Cooperation with the healthcare team (items 7 and 14) and Eating behavior (item, 17). As with the original construct, the analysis confirmed that item, 16 remained unsaturated in any subscale. A subsequent confirmatory factor analysis, steered by modification indices suggestions, culminated in a fitting overall model congruent with the original design [21].

The strengths of this study are that it was the first translation and validation of the DSMQ-R instrument for the Portuguese population with T2DM and that it was carried out with a relatively large sample. Limitations in this study pertained to the specific demographic characteristics of primary healthcare consultations, which predominantly involve individuals with T2DM. Those with type 1 diabetes mellitus or T2DM who are experiencing metabolic fluctuations are usually overseen by specialized endocrinology hospital units, hindering the validation of the DSMQ-R’s seven items [21–27] tailored for these subpopulations. Furthermore, logistical constraints prohibited the simultaneous collection of HbA1c values and questionnaire completion, leading to a potential six-month gap between these two data points. In future research, to minimize the possible gap between the HbA1C values and the DSMQ-R results, we suggested that a period should be established between the HbA1C collection and the completion of the questionnaire.

## Conclusions

Upon completing its translation and cross-cultural validation, the DSMQ-R instrument consistently demonstrated temporal stability and reliability, proving its appropriateness for use in the Portuguese population with T2DM. This tool effectively assesses diverse relevant self-care behaviors, as highlighted in its subscales. The measurement instrument may help detect suboptimal self-care practices and glycemic management, which may lead to diminished metabolic regulation and increased risks for serious diabetes complications.

## Data Availability

All data generated or analysed during this study are included in this published article.

## Abbreviations

ADA: American Diabetes Association
ANOVA: Analysis of variance
ARSLVT: Administração Regional de Saúde de Lisboa e Vale do Tejo
BMI: Body Mass Index
CFA: Confirmatory Factor Analysis
EFA: Exploratory factorial analysis
HbA1C: Glycated haemoglobin
SD: Standard deviation
T1DM: Type 1 diabetes mellitus
T2DM: Type 2 diabetes mellitus
